# Spontaneous rupture of a giant mature teratoma in the lateral ventricle: a case report

**DOI:** 10.3389/fonc.2024.1493982

**Published:** 2025-01-27

**Authors:** Yong Xiao, Liang Liu, Ran Wang, Dong Wang, Liangyuan Geng, Xinhua Hu, Yong Liu, Chunfa Qian, Yuanjie Zou

**Affiliations:** Department of Neurosurgery, Affiliated Nanjing Brain Hospital, Nanjing Medical University, Nanjing, China

**Keywords:** giant teratoma, rupture, the lateral ventricle, adolescent female, case report

## Abstract

We present the case of an adolescent female patient diagnosed with a ruptured lateral ventricle teratoma. Distinctive radiological and microscopic findings revealed floating oily globules within the ventricles and subarachnoid space. The spontaneous rupture of the teratoma may be attributed to age-dependent hormonal changes, which increase glandular secretion, cyst content volume, and intra-cystic pressure. The patient underwent gross total resection of the tumor, and the subsequent pathological examination confirmed the diagnosis of mature teratoma. Postoperatively, she was managed with routine follow-up without adjuvant chemotherapy or radiotherapy. During the 1-year follow-up period, the patient remained asymptomatic with no evidence of tumor recurrence. Based on this case, we recommend that gross total resection followed by close monitoring, without adjunctive chemotherapy or radiotherapy, can be an effective treatment strategy for patients with similar presentations.

## Introduction

Intracranial teratoma is a rare tumor that includes mature (considered benign) teratoma, immature teratoma, and teratoma with somatic-type malignancy, accounting for 0.4%–0.5% of all primary intracranial neoplasms (1, 2). Mature teratoma comprises adult-type tissue derived from all three germ cell layers (ectoderm, mesoderm, and endoderm), whereas immature teratoma consists of fetal tissue derived from the three embryonic layers. Prior studies have indicated that the majority of reported intracranial teratomas were congenital, defined as those appearing within the first 60 days of life (3, 4). Intracranial teratomas are primarily embedded in or near midline structures, such as the pineal region, suprasellar cistern, basal ganglia, thalamus, and third ventricle (5). Teratomas present during infancy and childhood, with male patients being affected more often than female patients (6).

While teratoma rupture has been described in various regions, such as the mediastinum, omental sac, pelvic cavity, and sacrococcygeal area (7), intracranial teratoma rupture has also been reported in locations including the pineal region, posterior fossa, frontal lobe, third ventricle, and fourth ventricle (8–10). We managed an adolescent female patient in whom a giant mass in the lateral ventricle and migrating oily globules in the cerebrospinal fluid (CSF) space were evident in computed tomography (CT) and magnetic resonance imaging (MRI) and were observed by surgical microscope. This case adds valuable insights into the clinical features, radiological findings, and therapeutic approaches for a spontaneously ruptured lateral ventricle teratoma.

## Case presentation

**Figure 1:**
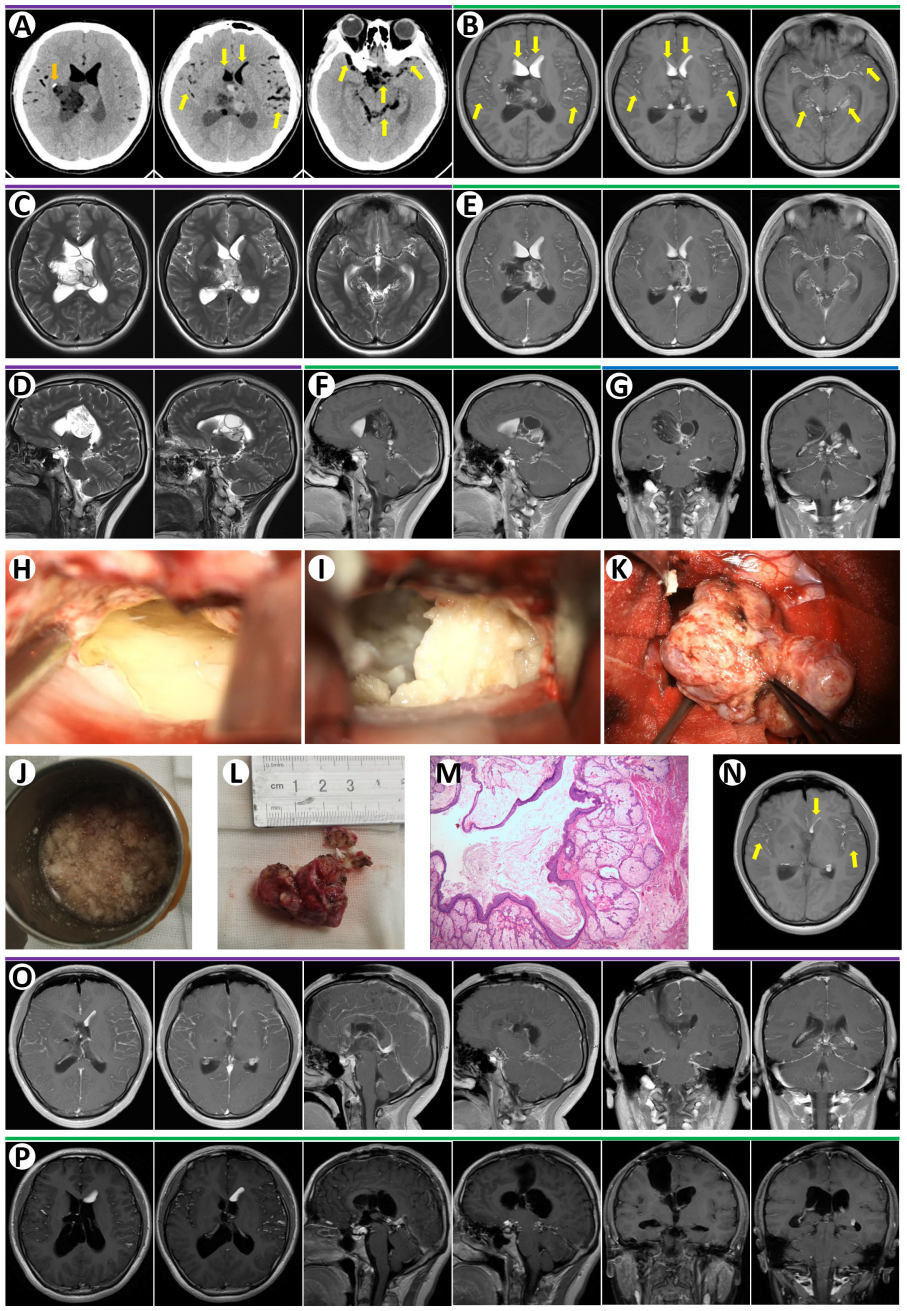
**(A)** Preoperative non-contrast-enhanced CT revealed a right lateral ventricle lesion with calcium in right rim (orange arrow). There were many low-density signals in bi-lateral ventricles and subarachnoid space likely oil or air (yellow arrows). **(B)** Axial T1-weighted MRI images demonstrated multiple hyperintense signals, oily globules, in ventricles and subarachnoid space (yellow arrows). Axial T2-weighted MRI images showed a lesion with mixed signal in the right lateral ventricle and septum pellucidum (**C**, axial; **D**, sagittal). T1-enhanced MRI images revealed heterogeneous enhancement of the lateral ventricle lesion and strong enhancement of septum pellucidum lesion (**E**, axial; **F**, sagittal; **G**, coronal). **(H)** The oily substances floating in CSF were observed during microsurgery. **(I)** The lesion containing cottage-cheese-like material was removed during microsurgery. **(K)** Gross total resection of the lesion during microsurgery. The oily substances in the cup **(J)** and the resected lesion **(L)** were demonstrated. **(M)** Paraffin section of this lesion confirmed mature teratoma. **(N)** Axial T1-weighted MRI image at day 1 after surgery revealed oily globules in ventricles and subarachnoid space (yellow arrows). **(O)** T1-enhanced MRI images at day 1 after surgery confirmed the gross total resection of the tumor. **(P)** T1-enhanced MRI images in 15 months after discharge showed no recurrence.

An 18-year-old female patient presented with a 9-day history of headache and vomiting. She did not have any history of trauma, and her medical history was unremarkable. Her physical examination revealed no abnormalities. Head CT showed a mixed-low-density lesion in the right lateral ventricle composed of fat components with an attenuation value of 30 HU. The solid part of the lesion was heterogeneous in density, with calcium in the right rim. Scattered oily substrates with an attenuation value of -110 HU were seen in the ventricles and subarachnoid space, suggesting tumor rupture (**Figure 1A**). The fat components in the anterior lateral ventricles and subarachnoid space were also hyperintense on T1-weighted images (**Figure 1B**) and hyperintense on T2-weighted images (**Figures 1C, D**). Fat suppression MRI was not performed preoperatively. MRI revealed a right lateral ventricle mass measuring 6 cm × 4 cm × 3.5 cm that extended to the septum pellucidum, causing obstructive hydrocephalus (**Figures 1C, D**). The lesion enhanced heterogeneously after contrast substance administration (**Figures 1E–G**). This imaging pattern suggested the rupture of a teratoma or dermoid cyst. Tumor marker evaluation was negative preoperatively, including Alpha Fetoprotein (AFP) (1.25 ng/mL), but without beta-Human Chorionic Gonadotropin (β-HCG) in our hospital laboratory examination protocol.

The patient then underwent right frontal craniotomy, with a percutaneous frontal lobe approach utilized. A C-shaped scalp incision was made on the right frontal region, exposing the area around the frontal coronal suture. A 5 cm × 4 cm free bone flap was created. The dura was cut in a C-shape and flipped toward the midline. A puncture was performed at the right lateral ventricle’s anterior horn, 3 cm lateral to the midline, and 1.5 cm anterior to the coronal suture. The ventricular needle was punctured approximately 5 cm into the subcortical matter, allowing CSF mixed with lipid-like droplets to flow out (**Figure 1H**). The white matter along the ventricular needle was aspirated, and the anterior part of the right lateral ventricle body was opened. It was noted that the right lateral ventricle was filled with lipid lesions and lipid-like fluids, which were removed in pieces. The lipid lesions had a cheese-like consistency (**Figures 1I, J**), and some softer parts contained glial components. The solid tumor exhibited expansive, multi-angled growth, pushing into the left lateral ventricle. Most of the solid tumor had clear boundaries, while some parts were indistinct from the ventricular walls, showing reactive gliosis. After complete separation of the tumor capsule, partial debulking of the intra-tumoral lipid cysts was performed. Following the electrocoagulation of the tumor feeding arteries, the solid tumor was entirely removed (**Figures 1K, L**) A video of the surgery for this patient is shown in **Supplementary Video S1**. Ventricular drainage was performed for 7 days postoperatively. Histopathological results confirmed a mature teratoma (**Figure 1M**). Postoperative day 1 cranial MRI confirmed complete tumor resection with no residual enhancing lesions (**Figures 1N, O**). The patient did not undergo chemotherapy or radiotherapy after discharge. She did not present any symptoms or recurrence of the tumor after a 15-month follow-up (**Figure 1P**).

## Discussion

### Pathogenesis

Two primary hypotheses can account for the pathogenesis of teratoma in the central nervous system. According to the germ cell theory, teratomas originate from primordial germ cells that migrate during embryogenesis and subsequently undergo malignant transformation, whereas the embryonic theory proposes that teratomas arise from pluripotent germ cells that have undergone aberrant migration (11, 12). Given that teratomas predominantly affect brain midline structures, and considering that the cerebral falx, cavernous sinus walls, and septum pellucidum share similar histological characteristics with the dura mater, it has been proposed that the aberrant migration of primordial germ cells into the dura mater might underlie the development of intracranial mature teratomas (12). This form of migration has also been proposed to be directed by a complex array of molecular events (11). Morphological alterations resulting from genetic modifications can play a crucial role in tumorigenesis. Several reported cases have shown that new teratomas can arise at alternative intracranial sites after the resection of the primary lesion, indicating that genetic factors might play a role in teratoma pathogenesis. The most frequently mutated somatic genes in intracranial teratomas were CARD11 and IRS1, each with a prevalence of 18%, followed by PSMD11, RELN, RRAS2, SMC1A, SYNE1, and ZFHX3, which exhibited mutation rates of 14% (13).

### Imaging features and differential diagnosis

The radiological features of teratoma depend mainly on the presence of the germinal layer. A characteristic CT feature is a low-density mass, including a markedly hypodense area containing fat. It has mixed heterogeneous signals on MRI, which reflects the varying content of tumor elements, including keratin and hyaline substances, oily substances, and dermal tissue. Sebaceous glands produce phospholipids, cholesteryl esters, and triglycerides, and rupture allows oily substances to disperse along the CSF space. Occasionally, the teratoma may be solid and homogeneous, implying poor differentiation, which tends to be immature or malignant. Cisternal fat globules demonstrate low attenuation on CT and may be mistaken for pneumocephalus. The lesions exhibited variable intensity on MRI, indicative of the underlying histological diversity, encompassing fibrosis, fatty tissue, calcification, cysts, and keratinocytes. Liu et al. (5) reported that among mature teratomas (n = 9), seven tumors exhibited non-enhancing multilocularity or heterogeneous enhancement of the cyst wall on contrast-enhanced T1-weighted images. Two tumors demonstrated moderate, heterogeneous enhancement within the solid components; in contrast, immature teratomas (n = 7) and those with malignant transformation (n = 2) showed heterogeneous, ring-like, or patchy intra-tumoral enhancement on contrast-enhanced T1-weighted images. MRI clearly distinguishes oily droplets and air bubbles within the subarachnoid space using fat-suppression sequences (14). However, the preoperative MRI of this patient did not incorporate a fat-suppression sequence. Because our case revealed a heterogeneous tumor including fat-associated signals in the ventricles and subarachnoid space, the oily globules likely originated from the tumor. Head tumors containing oily substances, such as teratomas and dermoid cysts, are difficult to differentiate based solely on radiological features, and a clear diagnosis should rely on pathological examination.

Since clinical outcomes in patients with intracranial teratomas depend on pathological results, differentiating mature teratoma from malignant teratoma is necessary. Allen et al. discovered that biomarkers like AFP and β-HCG can be used to differentiate between mature and malignant teratomas (15). These marker proteins can be detected in immature teratomas or embryonal carcinomas but are typically not expressed in mature teratomas. Other immunohistochemical markers, such as PLAP and c-kit, had limited utility in distinguishing between teratoma histological subtypes (16). Masao et al. measured the serum levels of AFP and β-HCG in seven patients with intracranial mature teratomas and found no elevated titers (17), consistent with our case.

### Clinical sequelae

Due to variations in tumor size and location, preoperative clinical symptoms differ among teratoma patients. A previous study demonstrated that gait disturbance is the predominant symptom, followed by headache and paroxysmal vomiting, likely due to intermittent increases in intracranial pressure in adult teratoma patients. However, the clinical context of teratoma rupture remains ambiguous. Released fat or oil can cause various sequelae such as headache, seizure, aseptic meningitis, or transient cerebral ischemia due to vasospasm (18). Chemical meningitis can be confused with bacterial meningitis, but CSF cultures are useful for differentiating between these conditions. According to a systematic review published in 2013, the administration of intravenous glucocorticoids may play a significant role in alleviating symptoms associated with chemical meningitis (19). Some researchers suggest irrigating the operative site with hydrocortisone to protect against aseptic meningitis; however, many note that postoperative aseptic meningitis is often transient and effectively managed with systemic dexamethasone therapy (20, 21). Our case presented with a sudden onset of headache and vomiting, suggesting a spontaneous rupture of the tumor.

### Reasons for rupture

The mechanism of spontaneous rupture is unclear. Most teratoma ruptures are induced by trauma, while spontaneous cases are rare. Lunardi and Missori stated that spontaneous rupture of cysts was correlated with head movements, particularly the pulsation of brain tissues (20). The water hammer effect could cause creaks in the cyst wall, leading to overflow of its contents. Given that the cyst wall has uneven thickness and different surrounding supportive structures, a teratoma could rupture when cyst content increases, as pressure at the cyst’s weak points rises. Furthermore, the combined mechanical stress of head injury, brain pulsation, and head movement may induce teratoma rupture. Stendel et al. proposed a hormone theory suggesting that age-dependent hormonal changes could lead to increased glandular secretion, thereby increasing cyst content and gradually elevating intra-cystic pressure, ultimately resulting in spontaneous rupture (22–24). Our adolescent female patient, with no history of trauma or surgery, fits this profile, indicating that age-dependent hormonal changes may have been a significant factor in the spontaneous rupture of her teratoma. Further investigation into the role of hormonal influences on cyst dynamics during adolescence could provide valuable insights into the mechanisms underlying this phenomenon.

### Therapeutic modality

Brain teratomas exhibit heterogeneous responses to treatments, and postoperative adjuvant treatment for brain teratomas has remained controversial due to the rarity of this tumor and the heterogeneity of histological subtypes. The 10-year survival rate for immature and malignant teratomas is 68%–70%, while for mature teratomas, it is 93% (25). Pure mature teratomas respond poorly to chemotherapy, and gross total resection is recommended for cure (25). However, no consensus has been reached on the use of radiotherapy for mature teratomas (26). While Ogawa et al. (27) and Sano (28) revealed that a combination of surgery and radiotherapy had a good curative effect on mature teratomas, Jakacki (29) and Selcuki et al. (30) argued that mature teratomas are not sensitive to radiotherapy, which could only cause brain trauma. Lagman et al. (31) advocated that mature teratomas are surgically curable and that radiotherapy is not the treatment of choice; however, relapsed mature teratomas are sensitive to radiotherapy. Therapeutic modalities for any teratoma should rely on histological classification and tumor marker levels. Our patient underwent gross total resection, and subsequent pathological results verified mature teratoma. Consequently, this patient was under follow-up without chemotherapy or radiotherapy after discharge.

## Conclusions

Our case highlighted the rare occurrence of spontaneous rupture of a lateral ventricle teratoma. The acute onset of symptoms and imaging confirmation of fatty elements were critical for diagnosing the teratoma. Differential diagnoses considered included other intraventricular masses such as ependymoma or astrocytoma; however, the presence of fat on imaging was highly indicative of a teratoma. Early surgical resection remained the primary treatment for benign cranial teratomas, even in cases of rupture, while adjuvant therapies like chemotherapy and radiotherapy were generally unnecessary. Further multicenter studies are needed to explore optimal treatment strategies for ruptured teratomas.

## Data Availability

The original contributions presented in the study are included in the article/**Supplementary Material**. Further inquiries can be directed to the corresponding author.

## Supplementary material

Supplementary Video 1 The related surgery video of the reported case was shown.
